# ATG5-mediated autophagy suppresses NF-κB signaling to limit epithelial inflammatory response to kidney injury

**DOI:** 10.1038/s41419-019-1483-7

**Published:** 2019-03-15

**Authors:** Xuan Peng, Yating Wang, Huiyan Li, Jinjin Fan, Jiani Shen, Xueqing Yu, Yi Zhou, Haiping Mao

**Affiliations:** 1 Department of Nephrology, the First Affiliated Hospital, Sun Yat-Sen University, Guangzhou, China; 2 NHC Key Laboratory of Nephrology, Guangzhou, China; 3 Guangdong Provincial Key Laboratory of Nephrology, Guangzhou, China

## Abstract

G2/M-arrested proximal tubular epithelial cells (TECs) after renal injury are linked to increased cytokines production. ATG5-mediated autophagy in proximal TECs has recently been shown to protect against G2/M cell cycle arrest and renal fibrosis. However, the impacts of autophagy in regulating inflammatorily response mounted by injured TECs remains largely unknown. In the present study, we investigated whether ATG5 acts as an innate immune suppressor in proximal TECs during kidney injury. Using the unilateral ureteric obstruction model in proximal tubule-specific autophagy-deficient mice, we demonstrated that ablation of epithelial *ATG5* genes markedly impaired autophagy, resulting in enhanced nuclear factor κB (NF-κB) activation, macrophage and lymphocyte infiltration, and proinflammatory cytokines production in obstructed kidneys, as compared with wild-type mice. Following stimulation with angiotensin II (Ang II), siRNA silencing of *ATG5* in cultured HK-2 cells or *ATG5*-deficient primary proximal TECs produced more cytokines, including IL-1β, IL-6, and TNF-α than did their control cells. Overexpressed ATG5, but not the autophagy-incompetent ATG5 mutant K130R in HK-2 cells, rendered resistant to Ang II-induced inflammatory response. Immunofluorescence assay indicated that ATG5 and p65 colocalized in the nucleus and cytoplasm, and their interaction was verified in immunoprecipitation assay from HEK-293T cell extracts. Genetic downregulation of endogenous *ATG5* increased Ang II-induced phosphorylation and nuclear translocation of p65 and transcriptional activity of NF-κB, whereas the overexpressed ATG5, rather than ATG5 mutant K130R, hampered activation of NF-κB signaling, suggest an autophagy-dependent anti-inflammatory effect of ATG5. Further, pharmacological manipulation of autophagy yielded similar results both in vivo and in vitro. Additionally, JSH-23, a specific inhibitor of NF-κB nuclear translocation, rescued Ang II-driven IL-1β production in *ATG5* siRNA-treated cells and decreased the proportion of cells in G2/M phase. In conclusion, ATG5-mediated autophagy in tubules targets NF-κB signaling to protect against renal inflammation.

## Introduction

Renal fibrosis is the result of the maladaptive repair and excessive inflammation in response to chronic injury, regardless of the underlying etiology. There is compelling evidence that under repeated and prolonged insults, not only immune cell, but also kidney intrinsic renal cells actively modulate immune reaction by releasing various proinflammatory cytokines^1^. These proinflammatory cytokines contribute to the recruiting of leucocytes into the kidneys. Although this process is a natural response, persistent and excessive inflammation leads to progressive kidney fibrosis and chronic kidney failure^2,3^. Tubular epithelium is a major site of cell injury and death during acute or chronic insults. Several studies have revealed that sustained injury causes renal tubular epithelial cell arrest in G2/M phase, which is associated with increased secretion of cytokines and pro-fibrotic factors^4–6^, suggesting the proinflammatory and fibrotic roles of tubular epithelial cells (TECs) in kidney injury. Therefore, understanding the effect of TECs in regulating the inflammatory milieu may develop a novel therapeutic strategy against renal fibrosis.

Autophagy, an evolutionarily conserved and genetically controlled pathway, has been considered as a homeostatic, catabolic degradation process to preserve cellular function^7,8^. Autophagy also serves as a stress-response pathway. Emerging evidence suggests that autophagy dysfunction contributes to several diseases, including cancer and autoimmunity, in which autophagy defects have a broad impact on innate and adaptive immune functions^7,9,10^. By using animal models with deletion of autophagy-related genes, autophagy has been implicated in protecting against kidney disease through maintaining tubule homeostasis and integrity, removal of damaged proteins, and regulation of production of cytokines and autoantibodies^11^. Our recent study demonstrated that ATG5-mediated autophagy in proximal TECs attenuated G2/M cell cycle arrest and renal fibrosis^6^, but the role of autophagy in regulating renal inflammation and the molecular mechanisms involved have not been yet determined.

Nuclear factor κB (NF-κB) is a transcriptional factor that participates in the modulation of inflammation, immunity, and cell fate. NF-κB activation has been documented in experimental and human renal inflammation as well as disease caused by infection, injury or autoimmune factors^12–14^. Blocking NF-κB activation ameliorates the progression of kidney injury, suggesting an important impact of NF-κB in the pathogenesis of kidney disease^15,16^. Recent studies have shown the interplay between autophagy and NF-κB signaling pathway in cancer and professional immune cells^17,18^. However, the role of NF-κB signaling, and the link to autophagy in regulating inflammatorily response mounted by injured TECs, has not been clarified.

In the present study, we demonstrated that the role of autophagy-related protein 5 (ATG5) in cell-autonomous defense against renal inflammation is autophagy-dependent in a model of renal fibrosis induced by unilateral ureteric obstruction (UUO). We also identified that ATG5-mediated autophagy suppressed inflammatory response via inhibition of NF-κB signaling.

## Results

### *ATG5* deficiency exacerbates renal inflammation in UUO mice model

**Fig. 1:**
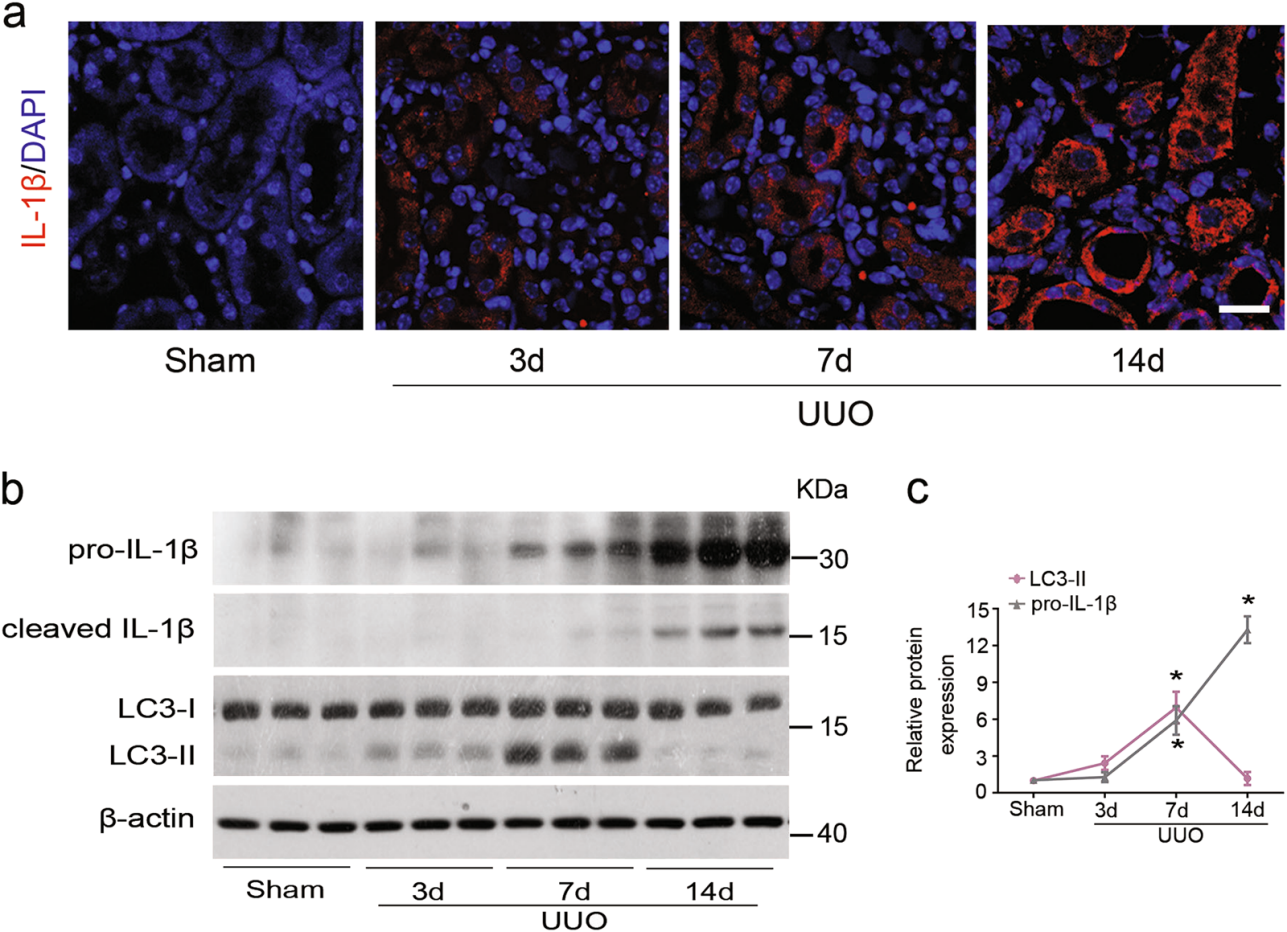
Autophagy and inflammation are induced successively in obstructed kidneys. C57Bl/6 mice were subjected to either sham operation or UUO. Mice were sacrificed at the indicated time. **a** Immunofluorescence staining for IL-1β (red) and DAPI (blue) on kidney sections. Scar bar: 20 μm. **b** Kidney tissue lysates were subjected to immunoblot analysis for pro-IL-1β, cleaved IL-1β and LC3. β-actin was used as a loading control. **c** Quantitative analysis of the ratio of LC3-II and pro-IL-1β to β-actin. Data are means ± SEM (*n* = 6 per group); *P < 0.05 vs. sham

Aberrant interstitial inflammation is associated with the development of kidney fibrosis^19^. We previously demonstrated that epithelial autophagy was active and ATG5-mediated autophagy exerted anti-fibrotic effect in experimental obstructive kidneys^6^. In this study, we aimed to investigate the role of autophagy in kidney inflammation. To this end, we first assessed dynamic events of both inflammation and autophagy in mice UUO kidneys, a model of progressive tubulointerstitial fibrosis. Immunofluorescence staining revealed that the expression of major proinflammatory factors such interleukin-1β (IL-1β) in TECs enhanced progressively over time with the obstruction and was most prominent in UUO kidneys at d 14, compared to sham-operated ones (Fig. 1a). Immunoblot analysis and densitometry verified the increased pro- and cleaved IL-1β expression in the kidney following UUO. Up-regulation of proinflammatory cytokines was accompanied by autophagy induction, which was a slight increase in the amount of LC3-II at d 3, peaking at d 7 and almost returning to basal levels at d 14 of UUO (Fig. 1b, c). These results demonstrated that autophagy and inflammation were induced during kidney injury.

**Fig. 2:**
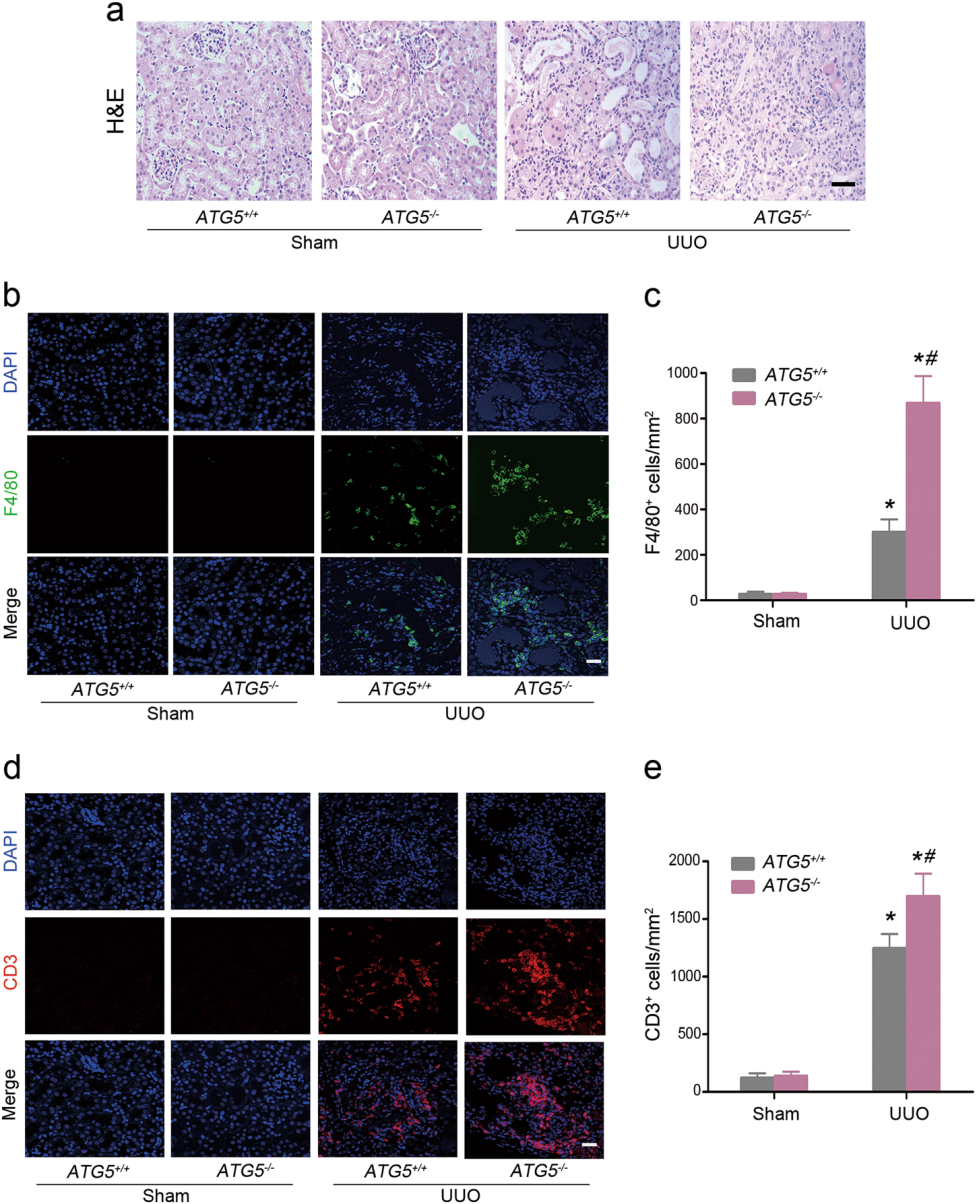
Autophagy deficiency enhances inflammatory cell infiltration during UUO. *ATG5*^*+/+*^ and *ATG5*^*−/−*^ mice were subjected to either sham operation or UUO, and kidney tissues were harvested after 7 days. **a** Representative H&E staining showed the morphologic injury and inflammatory cells infiltration. Scar bar: 20 μm. **b** Representative images of F4/80 staining (green) and DAPI (blue). Scale bar: 20 um. **c** Quantification of F4/80 positive cells. Data are expressed as mean ± SEM (*n* = 6); *P < 0.05 vs. sham; ^#^*P* < 0.01 vs. obstructed kidney of *ATG5*^+/+^ mice. **d** Representative images of CD3 staining (red) and DAPI (blue). Scar bar: 20 μm. **e** Quantification of CD3 positive cells. Data are expressed as mean ± SEM (*n* = 6); *P < 0.05 vs. sham; ^#^*P* *<* 0.01 vs. obstructed kidney of *ATG5*^+/+^ mice

Autophagy has been shown to function in diverse aspects of innate and adaptive immunity^10,20^. The protein ATG5 coded by the *ATG5* gene is essential for autophagosome formation. To assess the cell-type-specific effect of autophagy on interstitial inflammation in obstructive kidney, proximal tubular cell-specific *ATG5* knockout mice (*ATG5*^*−/−*^) and wild-type (*ATG5*^*+/+*^*)* mice were subjected to UUO^21^ and evaluated at d 7 after surgery. Hematoxylin-eosin staining revealed that UUO-induced leukocyte infiltration in the tubulointerstitium was markedly aggravated in the *ATG5*^*−/−*^ mice (Fig. 2a). Consistently, immunofluorescence staining confirmed dramatically elevated F4/80-positive macrophages (Fig. 2b, c) and CD3 positive lymphocytes (Fig. 2d, e) infiltration in the kidney cortex of *ATG5*^*−/−*^ mice, as compared with their *ATG5*^*+/+*^ littermates. Collectively, these data suggest that ATG5 exerts a protective role in UUO, whereas its deletion in tubular cells causes susceptibility to inflammatory response mounted by injured renal epithelium.

### *ATG5* deletion enhances cytokines production in TECs

**Fig. 3:**
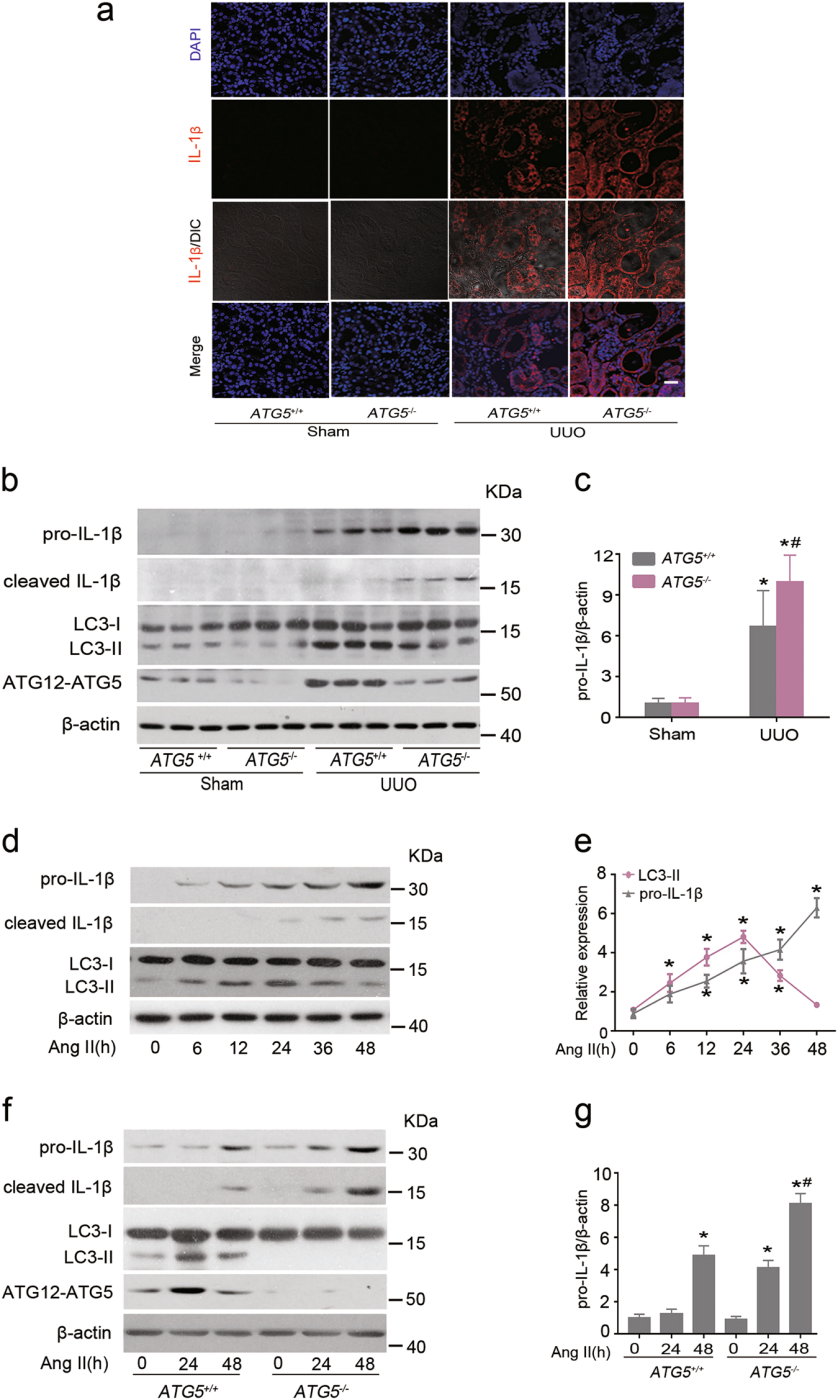
*ATG5* deletion in TECs increases IL-1β production. Animals and their treatment were the same as described in Fig. 2. **a** Representative immunofluorescence staining of IL-1β (red) in kidney sections. Scar bar: 20 μm. Cell nuclei were counterstained with DAPI (blue). **b** Representative images of immunoblot analysis of pro-IL-1β, cleaved IL-1β, LC3, ATG12–ATG5 kidney extracts. β-actin was used as a loading control. **c** Quantitative data of immunoblot for pro-IL-1β protein in kidney samples from the indicated groups of mice. Data are means ± SEM (*n* = 6); *P < 0.05 vs. sham; ^#^*P* < 0.01 vs. obstructed kidney of *ATG5*^+/+^ mice. **d** HK-2 cells were treated with 10^−6^ mmol/L of Ang II for the indicated time point. Cell lysates were subjected to immunoblot analysis for pro-IL-1β, cleaved IL-1β, and LC3. β-actin was used as a loading control. **e** Pro-IL-1β and LC3 contents were quantitatively analyzed using a densitometer. Values are mean ± SEM (*n* = 3); *P < 0.01 vs. control group. **f** Primary TECs from *ATG5*^+/+^ and *ATG5*^*−/−*^ mice were stimulated with 10^−6^ mmol/L of Ang II for 24 or 48 h. Cell lysates were probed with antibodies against the indicated proteins. **g** Densitometry of pro-IL-1β in immunoblots. Data are means ± SEM (*n* = 3); ^*^P < 0.05 vs. respective control; ^#^*P* < 0.05 vs. cells from *ATG5*^+/+^ TECs treated with Ang II for 48 h

Sublethal injury-driven renal tubular epithelial cell cycle arrest at G2/M is associated with provoking and exacerbating expression of inflammatory mediators^4^. However, it is unclear whether autophagy can function to modulate expression of proinflammatory cytokines in TECs. In line with the increased number of inflammatory cells accumulation at d 7 after UUO, kidneys from *ATG5*^*−/−*^ mice demonstrated significantly higher mRNA expressions of several proinflammatory cytokines, including IL-1β, interleukin-6 (IL-6) and tumor necrosis factor-α (TNF-α) when compared with those in *ATG5*^+/+^ mice (Supplemental Fig. 1a). Immunofluorescence staining revealed that the expression of IL-1β were substantially greater in proximal tubular cells of *ATG5*^−/−^ mice than those of *ATG5*^+/+^ mice (Fig. 3a). Western blot revealed that *ATG5* deletion significantly enhanced the expression of pro-IL-β and promoted IL-1β maturation (Fig. 3b, c). These results suggest that the innate immune response was greater in *ATG5*^*−/−*^ mice than in wild-type mice after UUO. Thus, we further determined the functional role of epithelial ATG5 in response to inflammatory stimuli in vitro. Normal human renal epithelial cell line HK-2 cells and primary proximal TECs isolated from 3-wk-old *ATG5*^*+/+*^ or *ATG5*^*−/−*^ mice were used and cultured in the presence or absence of angiotensin II (Ang II), an important mediator of tubulointerstitial inflammation. In HK-2 cells, Ang II treatment steadily increased pro-IL-1β protein expression over time and facilitated IL-1β maturation. This was accompanied by LC3-II up-regulation, which was the highest at 24 h and declined by 48 h (Fig. 3d, e). This was similar to the change of LC3-II in the kidney after UUO. Silencing *ATG5* expression potently increased Ang II-induced mRNA expression levels of IL-1β, IL-6, and TNF-α in cells exposed to Ang II but did not affect the basal levels of these cytokines (Supplementary Fig. 1b). Similarly, treatment of primary *ATG5*^*−/−*^ TECs with Ang II also increased mRNA expression of those cytokines (Supplementary Fig. 1c) as well as pro-IL-1β and cleaved IL-1β protein levels (Fig. 3f, g), when compared to primary *ATG5*^*+/+*^ TECs. Nonetheless, protein levels of LC3-II and ATG12–ATG5 conjugation were not obviously elevated in *ATG5*^*−/−*^ TECs treated with Ang II (Fig. 3f, g). Collectively, these findings suggest that ATG5 may act as an anti-inflammatory molecular to control the production of cytokines following insults.

### ATG5-dependent autophagy is essential to alleviate epithelial inflammatory responses

**Fig. 4:**
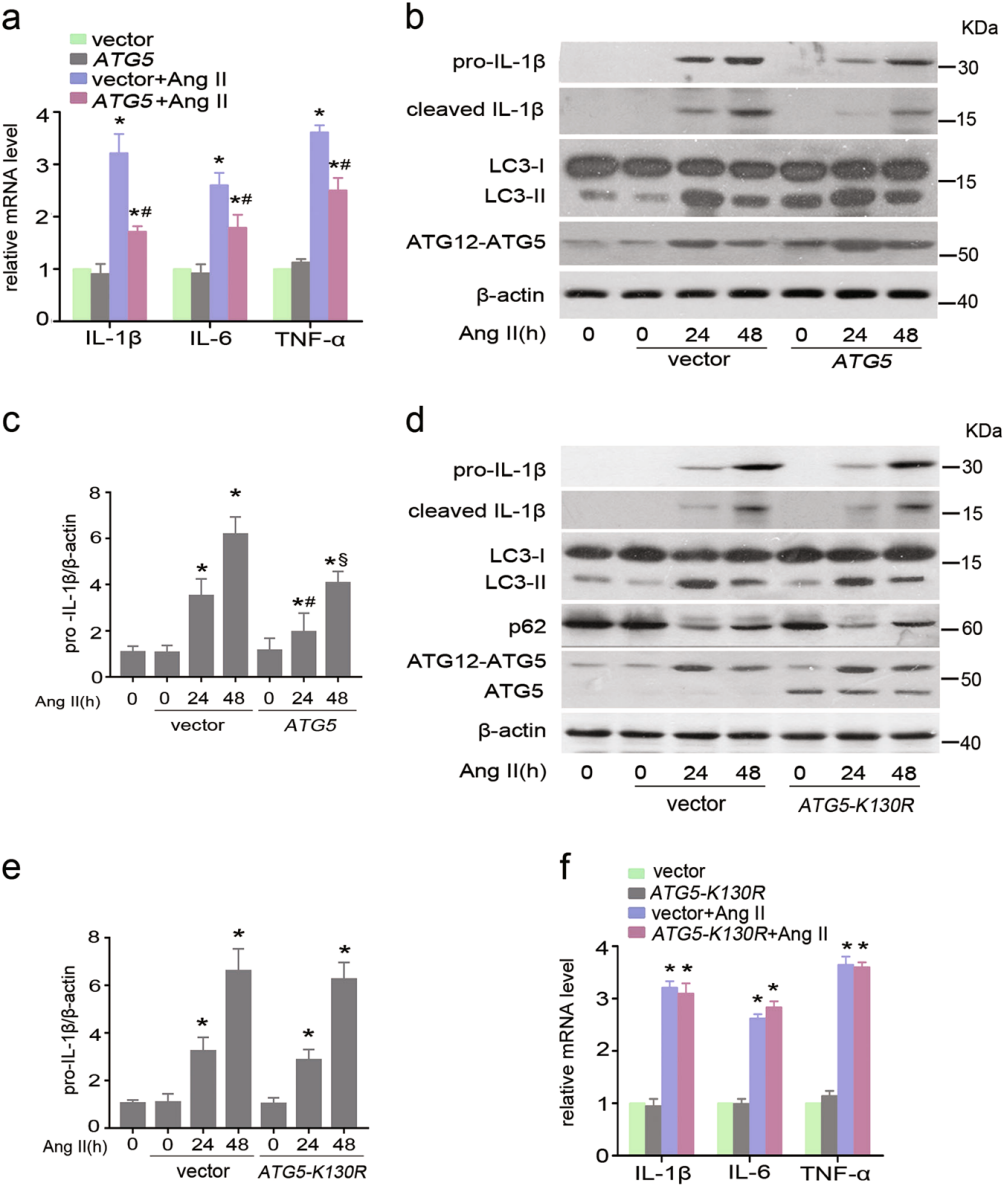
ATG5-mediated inhibition of Ang II-induced cytokines production is autophagy-dependent. HK-2 cells were transiently transfected with either pcDNA3.1-HA vector, pcDNA3.1-*ATG5* or pcDNA3.1-*ATG5*-K130R followed by stimulation with Ang II for the indicated time. **a** Cells were treated with Ang II for 24 h. Expression of IL-1β, IL-6, and TNF-α mRNA was determined by real-time PCR. GAPDH was the standard. Data are means ± SEM (*n* = 3); *P < 0.05 vs. Ang II-untreated cells; #*P* < 0.05 vs. Ang II-treated, empty vector-transfected cells. **b** Cell lysates were analyzed by immunoblotting with the indicated antibodies. **c** Densitometry of pro-IL-1β in immunoblots. Data are means ± SEM (*n* = 3); *P < 0.05 vs. Ang II-untreated cells; #*P* < 0.05 vs. Ang II-treated (24 h), empty vector-transfected cells, §*P* < 0.05 vs Ang II-treated 48 h, empty vector-transfected cells. **d** Representative immunoblots for the indicated proteins, with β-actin as loading control. **e** Densitometry of pro-IL-1β in immunoblots. Data are means ± SEM (n = 3); *P < 0.05 vs. Ang II-untreated cells. **f** Cells were treated with Ang II for 24 h. Expression of IL-1β, IL-6, and TNF-α mRNA was determined by real-time PCR. GAPDH was the standard. Data are means ± SEM (*n* = 3); *P < 0.05 vs. Ang II-untreated cells

ATG5, an essential component of the ATG12–ATG5 conjugation, is not only critical for autophagosome formation, but also regulates other cellular processes such as cell proliferation and apoptosis^22–24^. To determine whether ATG5 elicited immune regulation in TECs relies on autophagy function, HK-2 cells were individually transfected with plasmids expressing wild-type *ATG*5 or mutant *ATG*5 (*ATG5-K130R*), which is incapable of conjugation to ATG12 and loses autophagy function^25^, and the empty vector was used as control. Compared with empty vector, mRNA levels of IL-1β, IL-6 and TNF-α were dramatically reduced in ATG5-overexpressing cells upon exposure to Ang II for 24 h (Fig. 4a), which was coincident with increased LC3-II/LC3-I ratio and decreased protein levels of both pro- and cleaved IL-1β (Fig. 4b, c). In contrast, ectopic expression of dominant-negative mutant of *ATG5* (*ATG5-K130R*), notably enhanced protein levels of ATG5, but did not reverse Ang II-induced pro- and cleaved IL-1 β expression in HK-2 cells as compared with empty vector control (Fig. 4d, e). As predicted, there was no appreciable difference in mRNA levels of IL-1β, IL-6 and TNF-α mRNA between *ATG5-K130R* mutant transfected cells and empty vector control cells after Ang II exposure (Fig. 4f). Taken together, wild-type ATG5, but not autophagy-deficient mutant ATG5, reduced cytokines expression, suggesting that the anti-inflammatory effect of epithelial ATG5 is autophagy-dependent.

### *ATG5* deficiency promoted NF-κB activation in tubular cells

**Fig. 5:**
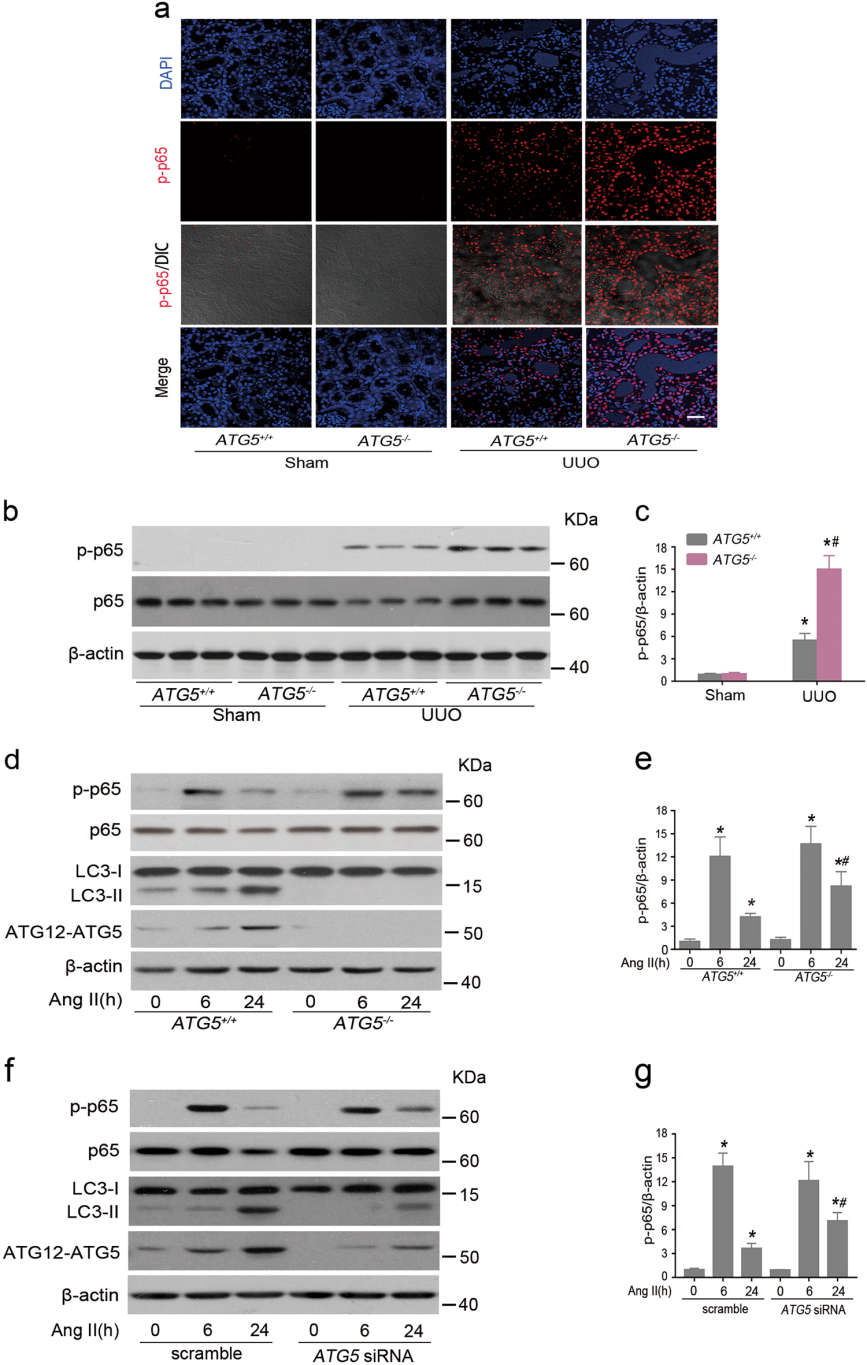
Downregulated *ATG5* expression provoked NF-κB activation. Animals and treatment were the same as described in Fig. 2. **a** Representative immunofluorescent staining of p-p65 (red) in kidney sections. Cell nuclei were counterstained with DAPI (blue). Scar bar: 20 μm. **b** Representative immunoblot analysis of p-p65 and p65 in kidney extracts. β-actin was used as a loading control. **c** Quantitative data of immunoblot for p-p65 protein in kidney samples from the indicated groups. Data are means ± SEM (*n* = 6); *P < 0.05 vs. sham; ^#^*P* *<* 0.01 vs. obstructed kidney of *ATG5*^+/+^ mice. **d** Primary TECs from *ATG5*^+/+^ and *ATG5*^*−/−*^ mice were stimulated with 10^−6^ mmol/L of Ang II for 6 or 24 h. Cell lysates were probed with antibodies against the indicated proteins. **e** Graphic representation of the relative abundance of p-p65 normalized with β-actin. Data are means ± SEM (*n* = 3); *P < 0.05 vs. Ang II-untreated corresponding TECs; #*P* < 0.05 vs. *ATG5*^+/+^ TECs treated with Ang II for 24 h. **f** HK-2 cells were transiently transfected with either scrambled or *ATG5* siRNA followed by treatment with Ang II for the indicated time point. Cell lysates were subjected to immunoblot analysis, β-actin was used as a loading control. **g** Relative expression levels of p-p65 normalized to β-actin. Data are means ± SEM (*n* = 3); *P < 0.05 vs. corresponding cells without Ang II treatment; #*P* < 0.05 vs. scrambled siRNA transfected cells with Ang II treatment for 24 h

NF-κB is one of the most important mediators of proinflammatory gene expression like IL-1β, IL-6, and TNF-α, and linked to inflammation-driven organ fibrosis^13^. Because autophagy is involved in NF-κB activation^26,27^, which has been demonstrated to participate in the immune responses in several diseases models^28,29^. We then examined the potential relationship between ATG5 and NF-κB signaling in the kidneys following UUO injury. Compared with sham-operated *ATG5*^*+/+*^ and *ATG5*^*−/−*^ mice, there were abundant positive phosphorylated p65 (p-p65) TECs at d 7 after UUO in *ATG5*^*+/+*^ animals, whereas this response was notably deteriorated in the renal tubules of *ATG5*^*−/−*^ mice (Fig. 5a). In agreement with these observations, immunoblotting analysis showed that *ATG5*^*−/−*^ mice had markedly higher levels of p-p65 than *ATG5*^*+/+*^mice in obstructed kidneys (Fig. 5b, c), suggesting that epithelial ATG5 is able to potentiate inactivation of NF-κB signaling in the kidney under this pathological condition. To determine the impact of ATG5 in NF-κB signaling in renal tubules, cells with or without *ATG5* downregulation were exposed to Ang II for monitoring the kinetics of NK-κB activation. We found that Ang II exposure led to phosphorylation of p65, which was detected as early as 15 min, peaked at 6 h and dropped gradually at 24 h (Supplemental Fig. 2). Based on this observation, the cells were treated with Ang II for 6 and 24 h in subsequent studies. The protein levels of p-p65 were dramatically increased in primary TECs from *ATG5*^*−/−*^ mice compared to those from *ATG5*^*+/+*^ mice after Ang II treatment for 24 h, despite there was a slight increase at 6 h with no statistically significance (Fig. 5d, e). A similar tendency was observed in HK-2 cells pretreated with specific *ATG5* siRNA (Fig. 5f, g). Notably, protein levels of ATG5 and LC3-II were downregulated concomitant with the increase of p-p65. Hence, ATG5 negatively regulates NF-κB activation in TECs.

Our previous study has connected autophagy with G2/M arrest, which is reportedly associated with NF-κB activation^30–32^, we thus characterized the interaction between NF-κB signaling and cell cycle progression. Immunofluorescent staining on kidney sections from UUO mice revealed an abundant expression of p-p65 in tubular cells, where a co-localization of p-p65 and p-H3 was noted (Supplementary Fig. 3a). IL-1β was also expressed mainly in p-H3 positive cells. In contrast, those proteins were barely detectable in kidneys from sham-operated animals (Supplementary Fig. 3b). Likewise, in the Ang II-stimulated HK-2 cells, p-p65 colocalized with p-H3, and enrichment of p-p65 appeared in the nucleus of p-H3-positive cells. No expression of p-H3 and p-p65 was observed in Ang II-untreated cells (Supplementary Fig. 3c). Moreover, flow cytometry analysis revealed that compared with vehicle-treated HK-2 cells, pretreatment with NF-κB nuclear translocation inhibitor JSH-23 reduced the proportion of cells in G2/M phase (Supplementary Fig. 3d). Collectively, these results suggest that inflammation-promoting function of NF-κB pathway is accompanied by promoting cell cycle arrest at G2/M phase in tubular cells, which may together contribute to inflammation in kidney injury.

### ATG5-mediated autophagy sequesters p65 protein in the cytoplasm to block NF-κB signaling

**Fig. 6:**
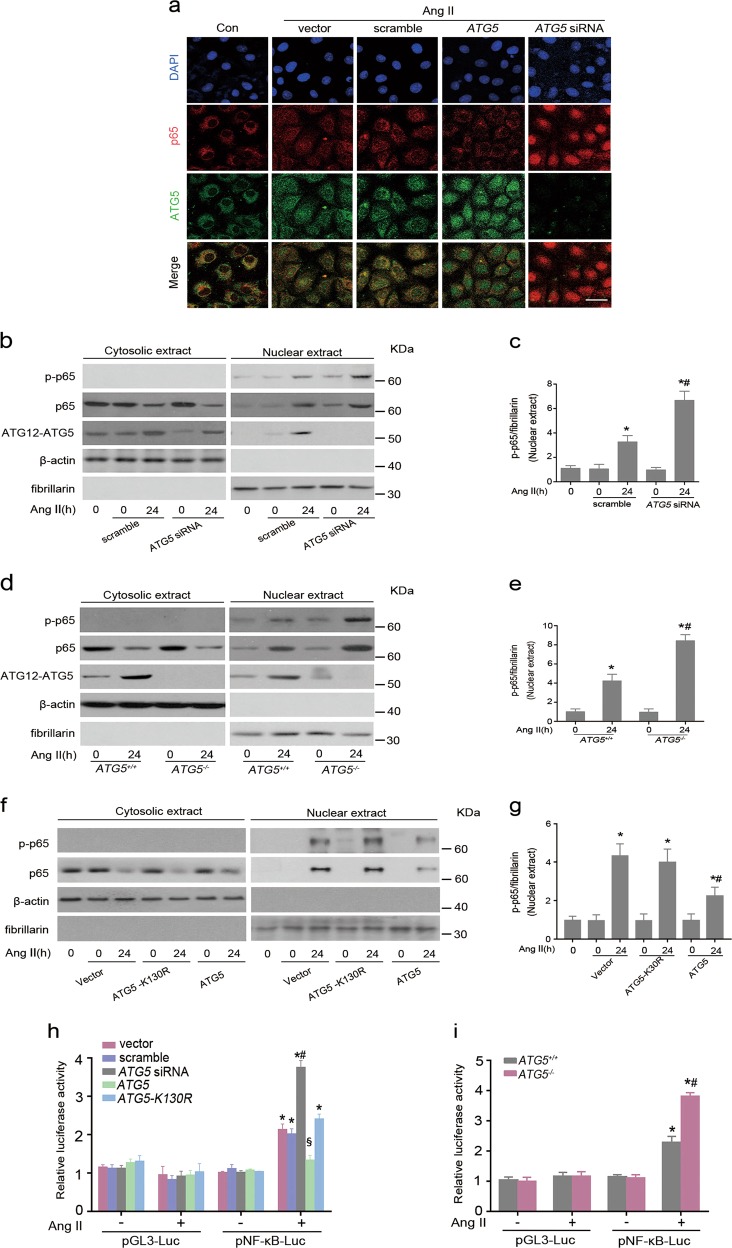
*ATG5* deficiency promotes NF-κB signaling. HK-2 Cells were transiently transfected with pcDNA3.1-HA vector, pcDNA3.1-*ATG5*, pcDNA3.1-*ATG5-K130R*, scrambled siRNA, or *ATG5* siRNA followed by incubation with Ang II for 24 h. **a** Immunofluorescence staining for p65 (red) and ATG5 (green). Cell nuclei were counterstained with DAPI (blue). Scar bar: 20 μm. **b** Immunoblot analysis of the amount of p-p65, p65 and ATG12–ATG5 expression in the cytosolic and nuclear fraction. β-actin and fibrillarin were respectively served as loading control for the cytosolic and nuclear proteins. **c** Quantitative determination of the relative abundance of p-p65 nuclear accumulation among different groups. Data are means ± SEM (*n* = 3); *P < 0.05 vs. Ang II-untreated cells; #*P* < 0.05 vs. Ang II-treated cells with scramble siRNA. **d** Immunoblot analysis of the indicated proteins in cytosolic and nuclear extracts of primary TECs from *ATG5*^*+/+*^ and *ATG5*^*−/−*^ mice. **e** Quantitative analysis of the relative abundance p-p65 in immunoblots. Data are means ± SEM (*n* = 3); *P < 0.05 vs. Ang II-untreated cells, #*P* < 0.05 vs. Ang II-treated *ATG5*^*+/+*^ TECs. **f** Immunoblot analysis of the amount of p-p65 and p65 levels in the cytosolic and nuclear fraction. **g** Quantitative determination of the relative abundance of nuclear p-p65 among different groups. Data are means ± SEM (*n* = 3); *P < 0.05 vs. Ang II-untreated cells; #*P* < 0.05 vs. Ang II-treated cells with ATG5-K130R. **h** HK-2 Cells were transiently transfected with either pGL3-Luc or pNF-κB-Luc luciferase reporter vector under the control of NF-κB responsible element, and then co-transfected with pcDNA3.1-HA vector, scramble siRNA, *ATG5* siRNA, pcDNA3.1-*ATG5*, or pcDNA3.1-*ATG5-K130R*. Cells were treated with Ang II for 24 h. pGL3-Luc served as a control. NF-κB transcriptional activities were determined by the luciferase reporter system in the indicated groups. Data are means ± SEM (*n* = 5); *P < 0.05 vs. pGL3-Luc and vector/scrambled-co-transfected cells with or without Ang II treatment; #*P* < 0.05 vs. pNF-κB-Luc and vector/scramble-cotransfected cells; §*P* < 0.05 vs. pNF-κB-Luc transfected cells with ATG5-K130R overexpression. **i** Primary *ATG5*^+/+^ and *ATG5*^*−/−*^ TECs transfected with either pGL3-Luc or pNF-κB-Luc vector followed by incubation with or without Ang II for 24 h, and luciferase activities were measured. Data are means ± SEM (*n* = 5); *P < 0.05 vs. pGL3-Luc transfected corresponding TECs with or without Ang II treatment; #*P* < 0.05 vs. pNF-κB-Luc transfected *ATG5*^*+/+*^ TECs

On the basis of the results above, we sought to explore the possible influence of ATG5 on p65 subcellular localization. As shown in Fig. 6a, in the absence of Ang II exposure, p65 and ATG5 were distributed and colocalized primarily within the cytoplasm in untreated HK-2 cells, whereas the localization of these two proteins was observed in the nucleus and the cytoplasm in Ang II-treated cells transfected with control. Overexpressed *ATG5* reduced nuclear translation of p65 after Ang II stimulation, which was accompanied by increased ATG5 both in the cytoplasm and in the nucleus. In contrast, downregulation of *ATG5* with siRNA caused the p65 protein to concentrate in the nucleus. To quantitatively verify these observations, p65 protein levels were assessed in cytoplasmic and nuclear fractions by immunoblotting analysis. Ang II treatment enhanced nuclear accumulation of p65 in scramble siRNA transfected cells compared to no-treatment controls. Nuclear translocation of p65 was even more pronounced in *ATG5* siRNA transfected cells (Fig. 6b, c). Comparable results were obtained by using primary TECs derived from *ATG5*^*+/+*^ and *ATG5*^*−/−*^ mice (Fig. 6d, e). However, autophagy-deficient ATG5 mutant K130R failed to reduce p65 nuclear accumulation (Fig. 6f, g), indicating the significance of ATG5-mediated autophagy in the repression of NF-κB signaling.

Since ATG5 mediates inhibition of p65 nuclear accumulation upon Ang II exposure, we further examined the role of ATG5 in NF-κB transcriptional activity by luciferase reporter gene assay. As shown in Fig. 6h, in the absence of Ang II treatment, basal NF-κB transcriptional activity was similar among all groups regardless of ATG5 status. Compared with pGL3-Luc control plasmid, the luciferase activity of pNF-κB-Luc was significantly increased in cells transfected with empty vector or siRNA scramble, and even greater in cells transfected with *ATG5* siRNA in response to Ang II stimulation. In contrast, ATG5 overexpression reduced NF-κB transcriptional activity, while this inhibitory effect was not observed in cells transfected with *ATG5-K130R* vector. Moreover, a concurrent increase of luciferase activity was verified in primary TECs derived from *ATG5*^*−/−*^ mice upon Ang II exposure compared to those from *ATG5*^*+/+*^ mice (Fig. 6i). Collectively, these data suggest that autophagy function is required for ATG5 blocking NF-κB signaling.

### ATG5 binds to p65 and inhibits NF-κB-mediated inflammatory responses

**Fig. 7:**
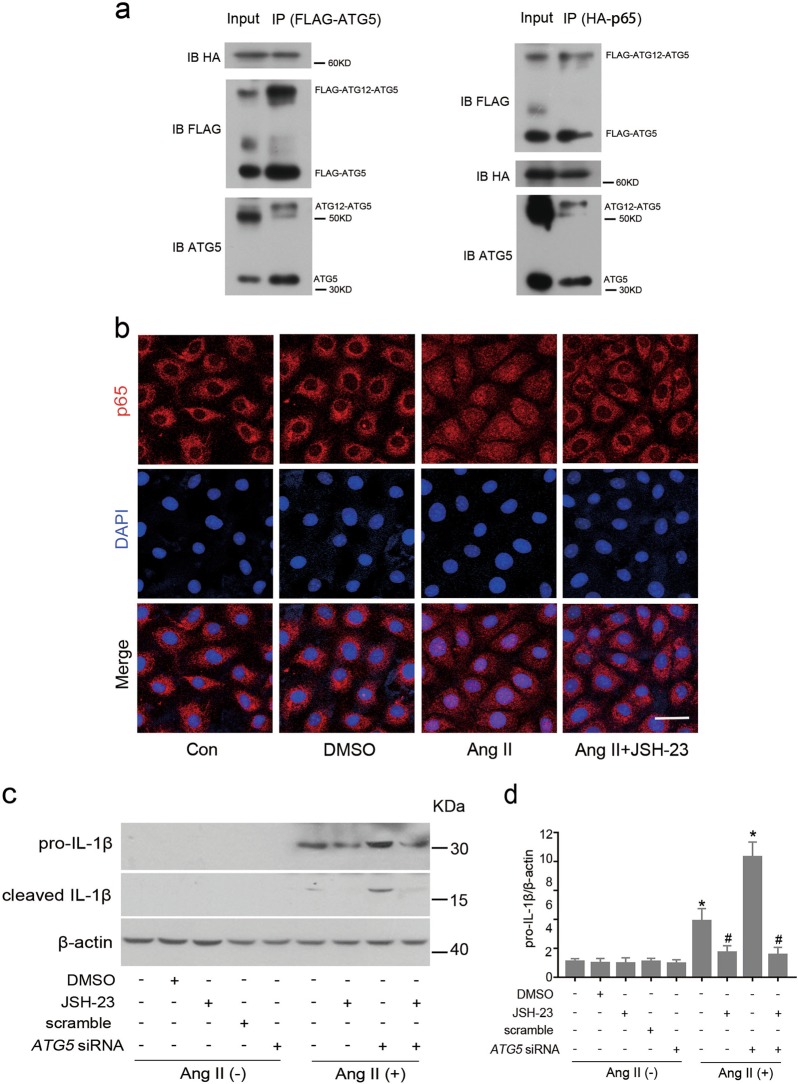
ATG5 interacts with p65 and prevents IL-β induction via inhibition of p65 nuclear translocation. **a** Co-immunoprecipitation of ATG5 with p65 in HEK-293T cells. FLAG-ATG5 and HA-p65 vectors were co-transfected into HEK-293T cells. Cell lysates were precipitated with anti-FlAG (left panel) or anti-HA (right panel) antibody-conjugated agarose, and blotted with anti-HA, anti-FLAG, and anti-ATG5 antibody as indicated. **b** HK-2 cells were pretreated with vehicle (DMSO) or 100 μM JSH-23, an inhibitor of nuclear translocation of NF-κB, and then transfected with scramble or *ATG5* siRNA followed by incubation with Ang II. Immunofluorescence staining for p65 (red). Cell nuclei were counterstained with DAPI (blue). Scar bar: 20 μm. **c** Immunoblot analysis of pro-IL-1β and cleaved IL-1β protein expression. **d** Relative expression levels of the pro-IL-1β normalized to β-actin by densitometry. Data are mean ± SEM (*n* = 3); *P < 0.05 vs. Ang II-untreated cells; #*P* < 0.05 vs. cells Ang II-treated alone

As ATG5 might interact with p65 (Fig. 6a), we next explored whether ATG5 forms a complex with p65. For this purpose, we co-expressed FLAG-tagged ATG5 and HA-tagged p65 in HEK-293 cells and performed the co-immunoprecipitation assay. The results showed that ATG5 was immunoprecipitated with p65 and vice versa (Fig. 7a), confirming that ATG5 is able to interact with p65 physically. However, the binding of ATG5 and p65 was not observed in HK-2 cells (data not shown). Thus, it is unknown if the interaction between two proteins is direct or dependent on the presence of other protein.

To clarify whether ATG5 deficiency-increased IL-1β production is dependent on NF-κB, JSH-23, a potent inhibitor of NF-κB nuclear translocation was used to treat HK-2 cells. Immunofluorescent staining showed that pretreatment with JSH-23 effectively blocked Ang II-induced nuclear p65 accumulation (Fig. 7b). Importantly, upon Ang II exposure, the increased expression of both pro- and cleaved IL-1β by *ATG5* siRNA was almost completely inhibited by JSH-23 pretreatment (Fig. 7c, d). Taking these data together, ATG5 appears to sequester p65 in the cytoplasm and suppress its nuclear translocation to inhibit inflammatory response in an NF-κB signaling-dependent manner.

### Pharmacologic manipulation of autophagy regulates NF-κB activation and cytokines production

**Fig. 8:**
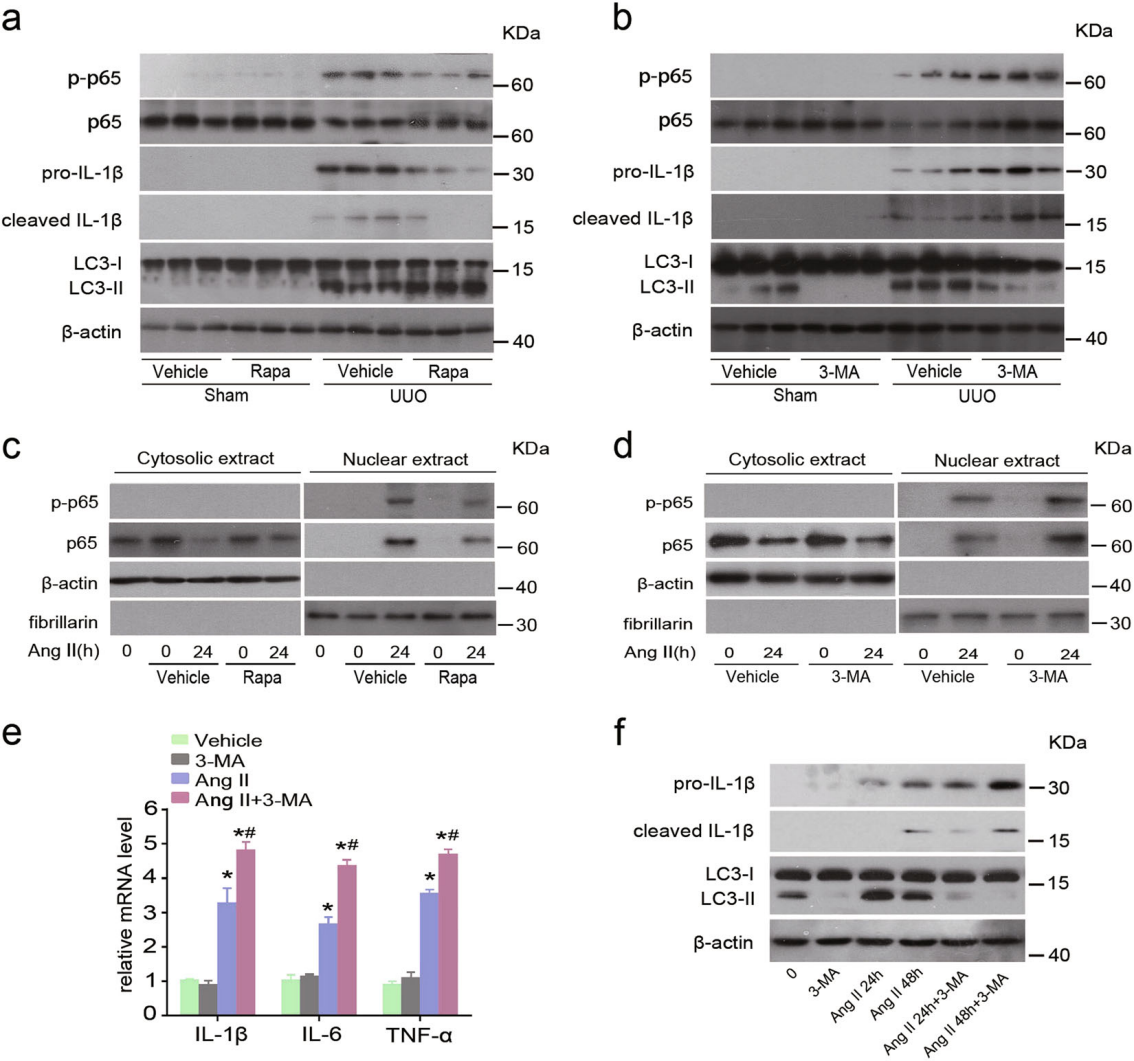
Pharmacologic induction of autophagy exerts a protective effect against NF-κB activation and cytokines production. **a** Immunoblot analyses of indicated proteins in the kidneys at day 7 after sham and UUO injury treated with or without rapamycin. **b** Immunoblot analyses of indicated proteins in the kidneys at day 7 after sham and UUO injury treated with or without 3-MA. **c** HK-2 cells were pretreated with vehicle or rapamycin prior to Ang II stimulation. Nuclear or cytoplasmic fractions were analyzed by immunoblotting with p-p65, p-65, fibrillarin (nuclear marker), and β-actin (cytoplasmic marker) antibodies. **d** HK-2 cells were pretreated with vehicle or 3-MA prior to Ang II stimulation. The amount of p-65 and p-p65 in the nuclear and cytoplasmic extracts was examined by immunoblot analysis. **e** The mRNA levels of IL-1β, IL-6 and TNF-α in HK-2 cells pretreated with vehicle or 3-MA followed by Ang II stimulation. Data are means ± SEM (*n* = 3). *P < 0.001 vs. Ang II-untreated cells; #*P* < 0.05 vs. Ang II-treated cells without 3-MA exposure. **f** Immunoblot analyses of indicated proteins among groups

To investigate the relationship between autophagy and inflammation further, we applied agonists or inhibitors to modulate autophagy. Compared with vehicle-treated mice, enhanced autophagy by rapamycin decreased p-65 phosphorylation and IL-1β production in the kidney of UUO mice (Fig. 8a). In contrast, inhibition of autophagy via 3-Methyladenine (3-MA) had opposite impacts on NF-κB activation and IL-1β expression (Fig. 8b). Consistently, rapamycin (Fig. 8c) attenuated, but 3-MA (Fig. 8d) increased Ang II-induced p-65 nuclear accumulation in HK-2 cells. Moreover, proinflammatory cytokines were downregulated by the 3-MA treatment at both mRNA (Fig. 8e) and protein (Fig. 8f) levels. Collectively, these data suggest an anti-inflammatory effect of autophagy during kidney injury.

## Discussion

Autophagy, as a host protective mechanism, plays a critical role in maintaining cellular homeostasis. G2/M-arrested TECs after renal injury are associated with increased cytokines production. We previously reported that ATG5-mediated autophagy in proximal TECs attenuated G2/M cell cycle arrest and inhibited renal fibrosis^4^. In this study, we aimed to explore the role of autophagy in renal inflammation and underlying potential molecular mechanisms. We found that proximal tubular epithelial cell-specific *ATG5*-deficient mice exhibited greater NF-κB activation, along with more leukocyte infiltration and cytokines production than wild-type littermates in the UUO model, suggesting an innate defense impact of autophagy in renal inflammation in response to injury. Further, in vitro study, ATG5 alleviated inflammatory response through inhibition of NF-κB signaling in Ang II-treated TECs, which was dependent on the functional role of autophagy. Our findings provide significant insight into the mechanism by which ATG5 suppresses renal inflammation and underline the importance of autophagy in mediating these renoprotective effects.

Prolonged renal inflammation triggered by acute or chronic insults contributes to the development of fibrosis at least partially through the increased leukocyte infiltration and the cytokines expression. Previous studies implicate a crucial role for tubular epithelial cells, especially those in G2/M phase of the cell cycle, in orchestrating renal inflammatory upon injury^4^. Autophagy has been reported to exert a protective effect in aspects of immunity and inflammation^33–35^. We previously showed that ATG5-mediated autophagy in proximal TECs attenuated G2/M cell cycle arrest and renal fibrosis. However, the interplay between autophagy in tubules and renal inflammation is not completely understood. In the present study, we demonstrated that both autophagy and inflammatory response were induced successively in obstructed kidney. Further, we found that autophagy deficiency by deletion of *ATG5* in proximal TECs resulted in dramatically increased leukocyte infiltration and proinflammation cytokines expression in obstructed kidneys. In vitro, overexpression of *ATG5* largely reduced Ang II-induced pro- and mature IL-1β expression in HK-2 cells and this inhibitory effect was eliminated in *ATG5* siRNA transfected HK-2 cells, as well as in primary TECs derived from *ATG5*^*−/−*^ mice. In agreement with our findings, studies in renal ischemia/reperfusion injury and toxic drugs revealed that pharmacological enhancement of autophagy reduced the production of proinflammatory cytokines^36,37^. However, Dong et al. showed that blockade of autophagy by conditional *ATG7* deletion from proximal TECs, unlike deletion of *ATG5*, suppressed interstitial macrophage infiltration and profibrotic cytokines production such as fibroblast growth factor 2, in obstructed kidneys^38^. The precise cause of the discrepancy between studies is unclear, but it may be due to the difference in the function of ATG5 and ATG7 in renal inflammation.

NF-κB signaling plays a key role in the inflammatory response during human and experimental kidney injury^39^. Previous studies have indicated a regulatory cross-talk between autophagy and NF-κB signaling cascade^26,27,40,41^. However, it is unclear about the function of autophagy in mediating NF-κB activation in renal TECs. In the present study, we noticed that compared to wild-type littermates, a significant increase of phosphorylated p-65 in obstructed kidneys of *ATG5*-deficient mice. Moreover, upon Ang II stimulation, primary TECs of *ATG5*^*−/−*^ mice and HK-2 cells transfected with *ATG5* siRNA showed enhanced p65 phosphorylation, nuclear translocation, and transcriptional activity, whereas overexpressed ATG5, rather than ATG5 mutant K130R inhibited p65 nuclear accumulation. On the other hand, *ATG5* knockdown exacerbated Ang II-driven production of pro- and cleaved IL-1β, which was reversed by an inhibitor of NF-κB nuclear translocation. All these findings suggest that ATG5 may negatively regulate the epithelial NF-κB signaling, thereby limiting renal inflammation. Notably, an interaction between ATG5 and p65 was characterized by colocalization analysis using immunofluorescence in HK-2 cells and co-immunoprecipitation in HEK-293 cells, but ATG5-p65 complex formation in HK-2 cells was not observed. Hence, the effect of ATG5 in sequestering p65 in the cytoplasm could be due to a direct interaction or to an indirect mechanism through a bridging molecule that somehow regulates p65. Although the previous study suggested that p65 was degraded by autophagy to terminate NF-κB activity^27^, study on the details of ATG5 regulatory functions, including p65 degradation remains to be explored in the future.

NF-κB activation has been connected with cell cycle progression^30^. In this study, an abundant expression of p-p65 in tubular cells co-localized with p-H3. Intriguingly, pretreatment with the specific NF-κB nuclear translocation inhibitor reduced the proportion of cells in G2/M phase. We previously demonstrated the role of ATG5-mediated autophagy in controlling G2/M cell cycle arrest^6^. This evidence, coupled with current results, suggest a synergism between ATG5-mediated autophagy and NF-κB activation in the context of G2/M cell cycle arrest, thereby cooperating to regulate kidney inflammation. The current study also revealed that overexpressed ATG5, but not the autophagy-incompetent ATG5-K130R mutant in HK-2 cells, decreased Ang II-induced NF-κB transcriptional activity and cytokines production, indicating that an activated autophagic capacity is essential for ATG5 to resist inflammation triggered by injured TECs. Further, pharmacological manipulation of autophagy yielded similar impacts in NF-κB activation and cytokines production. Yet, it is important to recognize that inhibition of mTOR by rapamycin has a modest effect on total protein synthesis^42^, which may also contribute to the observed downward trend of cytokines production. Collectively, these results verify the anti-inflammation effect of autophagy, which supports the notion that ATG5 protects against renal inflammation via an autophagy-dependent mechanism.

In conclusion, we demonstrate the critical role of ATG5 in regulating inflammation during the development of kidney fibrosis. Further, ATG5 inhibits inflammatory response mounted by injured TECs in an autophagy-dependent manner at least partially through blocking NF-κB signaling. Manipulation of autophagy could be a novel therapeutic approach for inflammation-derived kidney disease.

## materials and methods

### Reagents and antibodies

Angiotensin II and JSH-23 were purchased from Sigma-Aldrich (St. Louis, MO). The primary antibodies were obtained from the following sources: anti-LC3, anti-p-p65 NF-κB (Ser536), and anti-p65 NF-κB (Cell Signaling Technology, Beverly, MA), anti-ATG5, anti-IL-1β, anti-F4/80, anti-CD3, and anti-p-H3 (Abcam, Cambridge, MA); Anti-p62 GeneTex (Irvine, CA); anti-FLAG and anti-HA (Invitrogen Life Technologies, Paisley, UK); Alexa Fluor 488 goat anti-rabbit IgG antibody, Alexa Fluor 488 goat anti-mouse IgG antibody, Alexa Fluor 546 goat anti-mouse IgG antibody, and Alexa Fluor546 goat anti-rabbit IgG antibody (Invitrogen Life Technologies, Paisley, UK).

### Animal experiments

Wild-type C57BL/6 mice were obtained from the Sun Yat-Sen University Animal Center (Guangzhou, China). *ATG5*^flox/flox^ mice were purchased from the RIKEN BioResource Center (Tsukuba, Ibaraki, Japan). The *Kap-Cre* mice expressing Cre recombinase under the control of the Kap promoter were from the Jackson Laboratory (Bar Harbor, Maine, USA). Proximal tubule-specific *ATG5*^*−/−*^ mice have been described previously^6^. All mice were on a C57BL/6 background.

Animals were randomly assigned to sham operation or UUO. UUO surgery was performed as described previously^6^. Briefly, groups of male mice (*n* = 6 to 8 each) were anesthetized, and the left ureter was ligated twice with silk sutures. Sham-operated mice had their ureters exposed and manipulated without ligation. For pharmacological induction or suppression of autophagy, rapamycin (1 mg/kg) or 3-MA (30 mg/kg) were administered to mice through daily intraperitoneal (i.p.) injections, starting at the day before surgery until sacrifice on day 7. Both contralateral and obstructed kidneys were harvested at the end of study for histology, Western blotting, and real-time PCR analysis. All mice study was performed in accordance with the protocol approved by the Animal Care and Use Committee of the Sun Yat-Sen University.

### Histology and Immunostaining

Kidney histology was determined on formalin-fixed sections stained with hematoxylin and eosin. Immunofluorescence stainings were carried out for both kidney tissue and cultured cells following standard procedures, as previously described^6^. Samples were incubated with the indicated primary antibodies at 4 °C overnight, followed by their corresponding secondary antibodies for 1 h at room temperature. Nuclei were stained with DAPI. All images were collected by laser scanning confocal microscopy (Zeiss LSM 510 META; Carl Zeiss, Oberkochen, Germany).

Quantitation of immunostaining was carried on coded slides as previously described^43^. Briefly, 20 random and consecutive images were taken from the renal cortex under high-power fields (×400). The percentage of F4/80 or CD3-positive staining was extracted for intensity and analyzed with ImageJ software version 1.43j (NIH, Bethesda, MD). A mean area was calculated from the 20 images of an individual subject.

### Cell culture and treatment

Mouse TECs were isolated and cultured as described previously^6^. Briefly, kidney cortex from both *ATG5*^+/+^ and *ATG5*^*−/−*^ mice were harvested, dissected into small pieces, and then digested in a collagenase solution for 20 min at 37 °C. The supernatant was sieved through two nylon sieves with pore size 250 μm and 70 μm.

The primary TECs or the human kidney proximal tubule epithelial cells (HK-2) were cultured in DMEM/F-12 medium supplemented with 10% fetal bovine serum (Gibco, Grand Island, NY) and antibiotics. Cells were grown to approximately 80% confluence, serum-starved for 24 h and then treated with Ang II (10^−6^ mol/L) for different time periods. In some experiments, cells were pre-incubated with the inhibitor of p65 nuclear import, JSH-23 (100 μm/L) for 3 h before stimulation with Ang II.

### Plasmid Constructs and Transfection

The expression plasmids encoding *ATG5* wild type (pcDNA3.1-HA-*ATG5*, pcDNA3.1*-FLAG-ATG5*), *ATG5*-*K130R* mutant (pcDNA3.1-HA-*ATG5-K130R*) or *p65* (pcDNA3.1-*HA-p65*) were generated by PCR and confirmed by sequencing, as described previously^6^. The human *ATG5* siRNA and scramble siRNA products were purchased from Shanghai GenePharma Co., Ltd (Shanghai, China). HK-2 or HEK-293T cells were transiently transfected with various expression vector by using Lipofectamine 2000 reagent (Invitrogen, Carlsbad, CA) following the manufacturer’s protocol. The empty vector pcDNA3.1 or scramble siRNA was used as a mock transfection control. Overexpression or knockdown efficacy was assessed by Western blot analysis.

### Subcellular protein fractionation and immunoblotting

Nuclear and cytoplasmic fractions from cultured cells were prepared using the NE-PER Nuclear and Cytoplasmic Extraction kit (Thermo Fisher Scientific, Waltham, MA) following the manufacturer’s instructions. Cell or kidney cortex were lysed with RIPA buffer containing complete protease inhibitor cocktail (Roche, Basel, Switzerland). Lysates were centrifuged for 10 min at 4 °C, and the supernatants were obtained. The protein of extracts was subjected to Western blot analysis using the method described previously^6^. Fibrillarin (a nuclear protein) and β-actin were used as loading controls.

### FLAG or HA-tagged protein immunoprecipitation

The FLAG or HA-tagged protein was immunoprecipitated by using anti-FLAG or anti-HA M2-Agarose kit following the manufacturer’s instructions. Briefly, HEK-293T cells were cultured in 100 mm dish followed by transfection treatments, cells were lysed in immunoprecipitation lysis buffer, followed by incubation on ice for 15 min. Cell lysates were then centrifuged at 12,000 × *g*, for 10 min at 4 °C and supernatants were collected. For every 1 ml lysates, 40 μl of agarose beads were added for precipitating tagged protein, and samples were incubated in 4 °C by a roller shaker overnight. After being washed, immunoprecipitated proteins were analyzed by Western blot.

### RNA isolation and real-time PCR analysis

Total RNA was isolated from either the kidney tissues or the cultured cells using TRIzol reagent (Invitrogen, Carlsbad, CA), and then reversely transcribed into cDNA using Transcriptor First Strand cDNA Synthesis Kit (Roche, Basel, Switzerland) according to the manufacturer’s instructions. Real-time PCR was performed on the ABI Prism 7900 sequence detection system (Applied Biosystems, Foster City, CA) using SYBR Green PCR Master Mix and gene-specific primers. Human primers used in this study were as follows: IL-1β: forward, 5′-ATGATGGCTTATTACAGTGGCAA-3′, reverse, 5′-GTCGGAGATTCGTA GCTGGA-3′; IL-6: forward, 5′-ACTCACCTCTTCAGAACGAATTG-3′, reverse, 5′-CCATCTTTGGAAGGTTCAGGTTG-3′, TNF-α: forward, 5′-CCTCTCTC TAATCAGCCCTCTG-3′, reverse, 5′-GAGGACCTGGGAGTAGATGAG-3′, GAPDH: forward,5′-GCACCGTCAAGGCTGAGAAC-3′, reverse, 5′-TGGTGAAGACGCCAGTGGA-3′. Mouse primer sequences were showed below: IL-1β: forward, 5′-GAAATGCCACCTTTTGACAGTG-3′, reverse, 5′-TGGATGCTCTCATCAGGACAG-3′; IL-6: forward, 5′-CTGCAAGAGACTTCC ATCCAG-3′, reverse, 5′-AGTGGTATAGACAGGTCTGTTGG-3′; TNF-α: forward, 5′-CCTGTAGCCCACGTCGTAG-3′, reverse, 5′-GGGAGTAGACAAGGT ACAACCC −3′; GAPDH: 5′-ATGTTCCAGTATGACTCCACTCACG-3′, reverse, 5′-GAAGACACCAGTAGACTCCACGACA-3′. GAPDH was used as an internal control and the ratio of the mRNA examined to the GAPDH was calculated.

### Dual-luciferase assay

NF-κB transcriptional activity was examined by using NF-κB-Luc luciferase reporter gene assay. Cells were transiently cotransfected with pNF-κB-Luc and pRL-TK, a control for transfection efficiency (Promega, Madison, WI) using Lipofectamine 2000 transfection reagent (Invitrogen, Carlsbad, CA), following the manufacturer’s protocol. The pGL3-Luc plasmid without carrying NF-κB responsible DNA elements was used as a control. After 6 h of transfection, cells were incubated with complete medium for 24 h followed by the indicated treatments. Luciferase activities were evaluated using the Dual-Luciferase Reporter Assay System (Promega) according to the manufacturer’s instructions. The activity was normalized as to transfection efficiency using the Renilla luciferase activity of pRL-TK. Experiments were performed in triplicate and at least five independent experiments.

### Cell cycle analysis

Propidium iodide was used to determine the cell cycle distribution as previously reported^6^. After treatment with Ang II or JSH23, HK-2 cells were washed with PBS, harvested, and fixed with 70% ethanol at 4 °C overnight. Cells were resuspended in PBS and stained with 40 μg/ml PtdIns for 30 min on ice, followed by flow cytometry analysis.

### Statistical analysis

The data are expressed as mean ± SEM. Student’s t-test was used for comparisons between two groups; one-way analysis of variance (ANOVA) followed by Tukey’s post-test for multiple comparisons was used to determine significant differences among groups of three or more. *P* values < 0.05 were considered statistically significant.

## Supplementary information

supplemental figure 1

supplemental figure 2

supplemental figure 3

Supplemental Figure legends

## References

[1] Liu BC, Tang TT, Lv LL, Lan HY (2018). Renal tubule injury: a driving force toward chronic kidney disease. Kidney Int..

[2] Meng XM, Nikolic-Paterson DJ, Lan HY (2014). Inflammatory processes in renal fibrosis. Nat. Rev. Nephrol..

[3] Cantaluppi V (2014). Interaction between systemic inflammation and renal tubular epithelial cells. Nephrol., Dial., Transplant..

[4] Yang L, Besschetnova TY, Brooks CR, Shah JV, Bonventre JV (2010). Epithelial cell cycle arrest in G2/M mediates kidney fibrosis after injury. Nat. Med..

[5] Mao H (2008). HSP72 attenuates renal tubular cell apoptosis and interstitial fibrosis in obstructive nephropathy. Am. J. Physiol. Ren. Physiol..

[6] Li H (2016). Atg5-mediated autophagy deficiency in proximal tubules promotes cell cycle G2/M arrest and renal fibrosis. Autophagy.

[7] Zhong Z, Sanchez-Lopez E, Karin M (2016). Autophagy, inflammation, and immunity: a troika governing cancer and its treatment. Cell.

[8] Jiang P, Mizushima N (2014). Autophagy and human diseases. Cell Res..

[9] De Meyer GR (2015). Autophagy in vascular disease. Circ. Res..

[10] Deretic V (2015). Immunologic manifestations of autophagy. J. Clin. Invest..

[11] Lenoir O, Tharaux PL, Huber TB (2016). Autophagy in kidney disease and aging: lessons from rodent models. Kidney Int..

[12] Zhang H, Sun SC (2015). NF-kappaB in inflammation and renal diseases. Cell Biosci..

[13] Ray KLiver (2012). Activation of NF-kappaB signaling in hepatocytes induces liver fibrosis.. Nat. Rev. Gastroenterol. Hepatol..

[14] Zhong Z (2016). NF-kappaB restricts inflammasome activation via elimination of damaged mitochondria. Cell.

[15] Marko L (2016). Tubular Epithelial NF-kappaB activity regulates ischemic AKI. J. Am. Soc. Nephrol.: JASN.

[16] Choi M (2017). Endothelial NF-kappaB Blockade Abrogates ANCA-Induced GN. J. Am. Soc. Nephrol.: JASN.

[17] Liu K (2018). SKP2 attenuates NF-kappaB signaling by mediating IKKbeta degradation through autophagy. J. Mol. Cell Biol..

[18] Zhang Y (2017). ANGPTL8 negatively regulates NF-kappaB activation by facilitating selective autophagic degradation of IKKgamma. Nat. Commun..

[19] Lv W, Booz GW, Wang Y, Fan F, Roman RJ (2018). Inflammation and renal fibrosis: Recent developments on key signaling molecules as potential therapeutic targets. Eur. J. Pharmacol..

[20] Ma Y, Galluzzi L, Zitvogel L, Kroemer G (2013). Autophagy and cellular immune responses. Immunity.

[21] Kimura T (2011). Autophagy protects the proximal tubule from degeneration and acute ischemic injury. J. Am. Soc. Nephrol.: JASN.

[22] Maskey D (2013). ATG5 is induced by DNA-damaging agents and promotes mitotic catastrophe independent of autophagy. Nat. Commun..

[23] Cianfanelli V (2015). AMBRA1 links autophagy to cell proliferation and tumorigenesis by promoting c-Myc dephosphorylation and degradation. Nat. Cell Biol..

[24] Zhang D (2017). Protein kinase cdelta suppresses autophagy to induce kidney cell apoptosis in cisplatin nephrotoxicity. J. Am. Soc. Nephrol.: JASN.

[25] Mizushima N (1998). A protein conjugation system essential for autophagy. Nature.

[26] Kanayama M (2015). Autophagy enhances NFkappaB activity in specific tissue macrophages by sequestering A20 to boost antifungal immunity. Nat. Commun..

[27] Chang CP, Su YC, Hu CW, Lei HY (2013). TLR2-dependent selective autophagy regulates NF-kappaB lysosomal degradation in hepatoma-derived M2 macrophage differentiation. Cell Death Differ..

[28] Djavaheri-Mergny M, Codogno P (2007). Autophagy joins the game to regulate NF-kappaB signaling pathways. Cell Res..

[29] Yang S, Qiang L, Sample A, Shah P, He YY (2017). NF-kappaB signaling activation induced by chloroquine requires autophagosome, p62 protein, and c-jun n-terminal kinase (JNK) signaling and promotes tumor cell resistance. J. Biol. Chem..

[30] Joyce D (2001). NF-kappaB and cell-cycle regulation: the cyclin connection. Cytokine Growth Factor Rev..

[31] Liang Z (2015). HIV-1 Vpr protein activates the NF-κB pathway to promote G2/M cell cycle arrest. Virol. Sin..

[32] Cude K (2007). Regulation of the G2–M cell cycle progression by the ERK5– NFκB signaling pathway. J. Cell Biol..

[33] Kimura T, Isaka Y, Yoshimori T (2017). Autophagy and kidney inflammation. Autophagy.

[34] Fougeray S, Pallet N (2015). Mechanisms and biological functions of autophagy in diseased and ageing kidneys. Nat. Rev. Nephrol..

[35] Zhou XJ, Zhang H (2012). Autophagy in immunity: implications in etiology of autoimmune/autoinflammatory diseases. Autophagy.

[36] Ling H (2016). The Effect of autophagy on inflammation cytokines in renal ischemia/reperfusion injury. Inflammation.

[37] Zhang LX (2017). Niclosamide attenuates inflammatory cytokines via the autophagy pathway leading to improved outcomes in renal ischemia/reperfusion injury. Mol. Med. Rep..

[38] Livingston MJ (2016). Persistent activation of autophagy in kidney tubular cells promotes renal interstitial fibrosis during unilateral ureteral obstruction. Autophagy.

[39] Sanz AB (2010). NF-kappaB in renal inflammation. J. Am. Soc. Nephrol..

[40] Trocoli A, Djavaheri-Mergny M (2011). The complex interplay between autophagy and NF-kappaB signaling pathways in cancer cells. Am. J. Cancer Res..

[41] Baldwin AS (2012). Regulation of cell death and autophagy by IKK and NF-kappaB: critical mechanisms in immune function and cancer. Immunol. Rev..

[42] Iadevaia V, Huo Y, Zhang Z, Foster LJ, Proud CG (2012). Roles of the mammalian target of rapamycin, mTOR, in controlling ribosome biogenesis and protein synthesis. Biochem. Soc. Trans..

[43] Zhou Y (2016). Heat shock protein 72 antagonizes STAT3 signaling to inhibit fibroblast accumulation in renal fibrogenesis. Am. J. Pathol..

