# Supplementary figures and images for: ATG5-mediated autophagy suppresses NF-κB signaling to limit epithelial inflammatory response to kidney injury
Source: Cell Death Dis. 2019 Mar 15;10(4):253. doi: 10.1038/s41419-019-1483-7 (PMC6420665; doi:10.1038/s41419-019-1483-7)

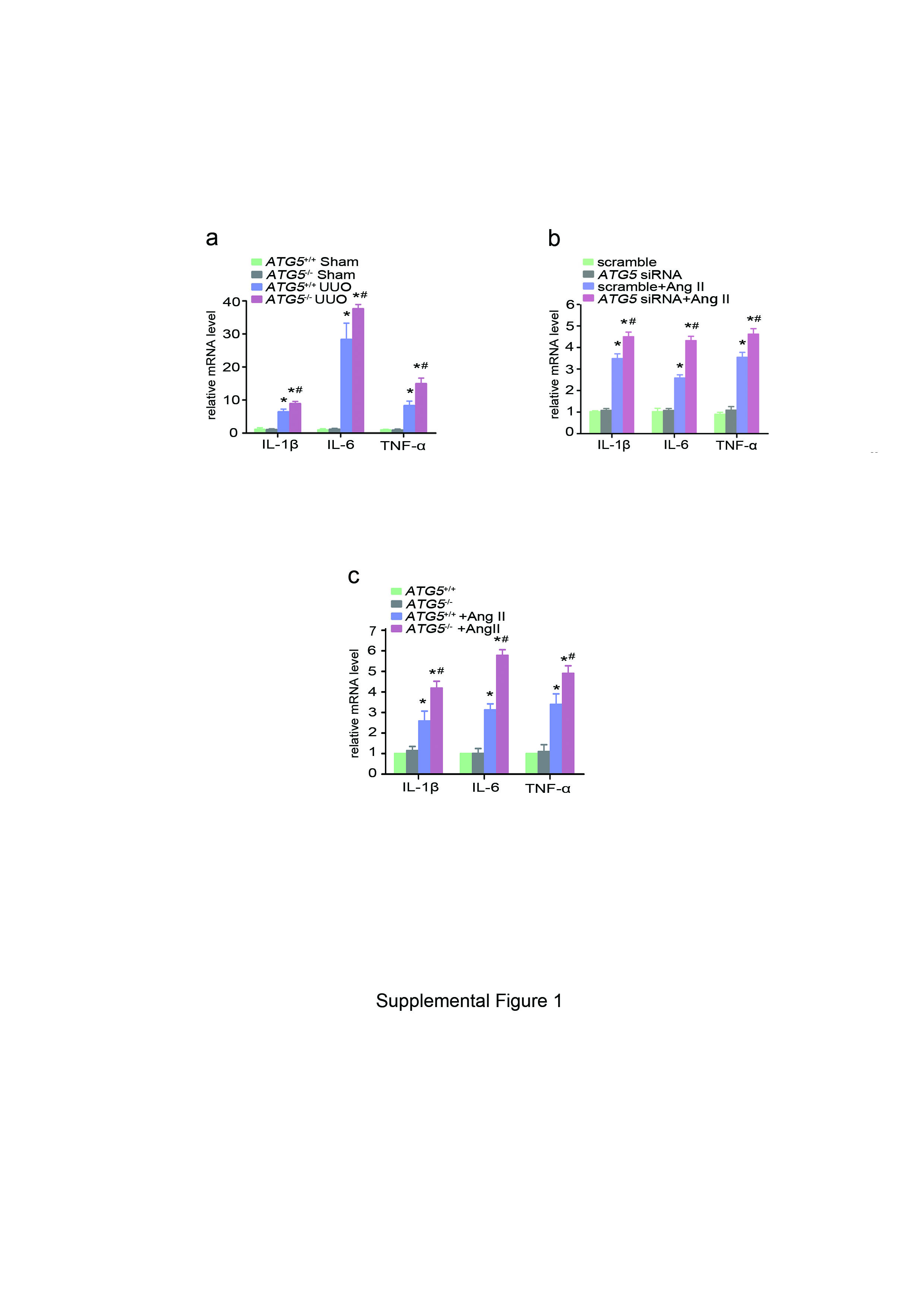

Supplement: Supplementary file 1 — supplemental figure 1 [file 41419_2019_1483_MOESM1_ESM.jpg]

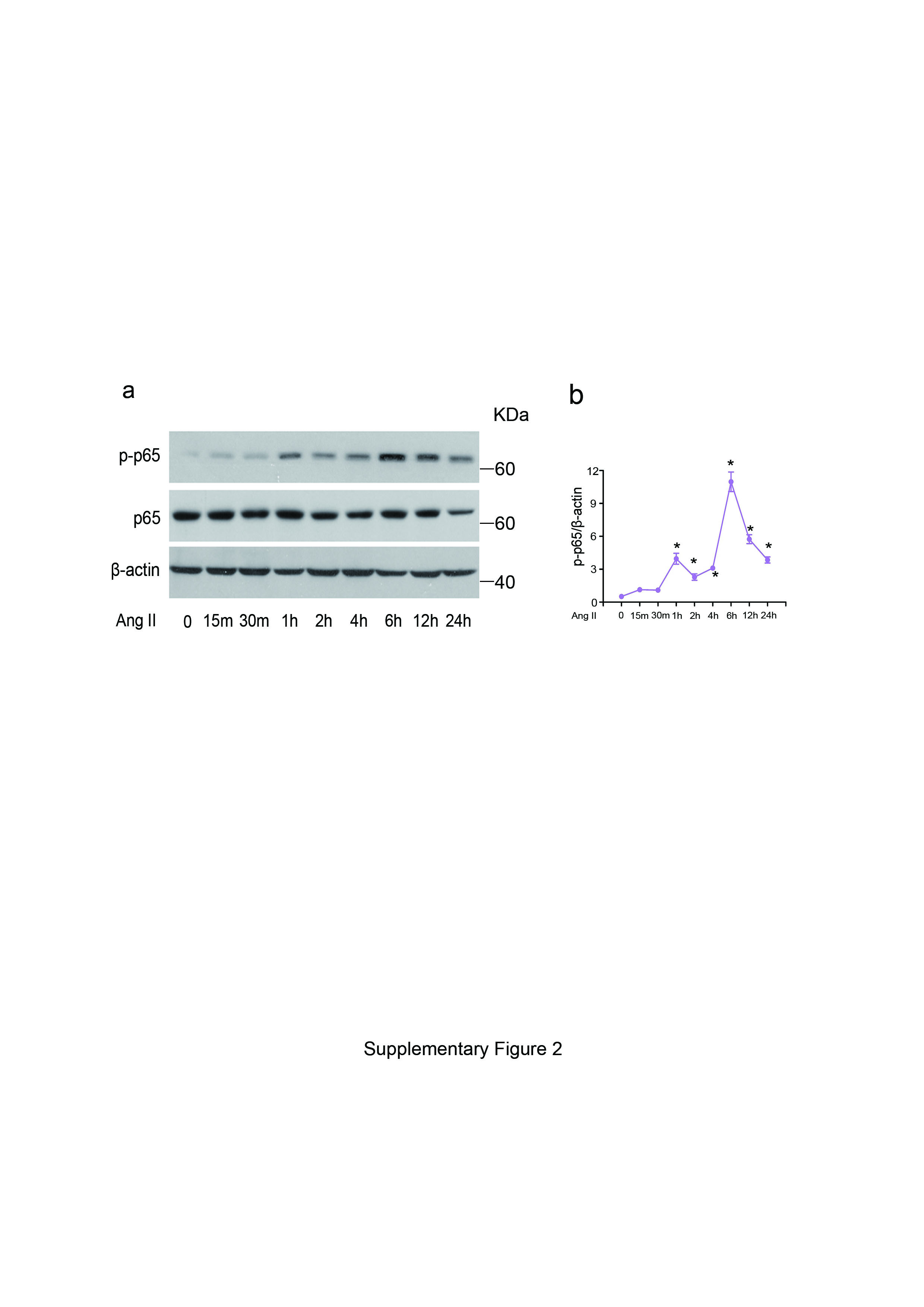

Supplement: Supplementary file 2 — supplemental figure 2 [file 41419_2019_1483_MOESM2_ESM.jpg]

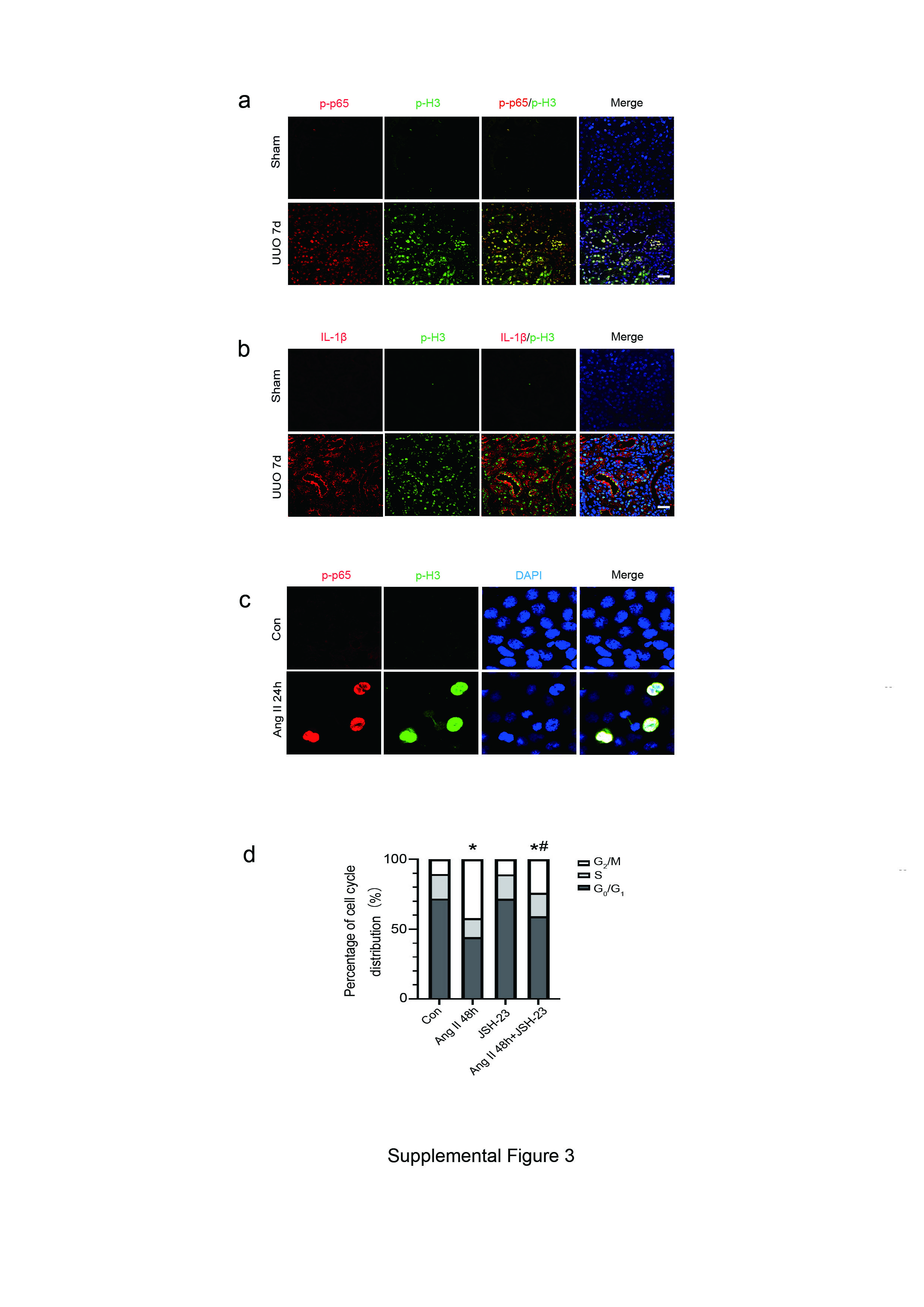

Supplement: Supplementary file 3 — supplemental figure 3 [file 41419_2019_1483_MOESM3_ESM.jpg]
