# Supplementary material for: ATG5-mediated autophagy suppresses NF-κB signaling to limit epithelial inflammatory response to kidney injury
Source: Cell Death Dis. 2019 Mar 15;10(4):253. doi: 10.1038/s41419-019-1483-7 (PMC6420665; doi:10.1038/s41419-019-1483-7)
Supplement: Supplementary file 4 — Supplemental Figure legends [file 41419_2019_1483_MOESM4_ESM.docx]

**ATG5-mediated autophagy suppresses NF-κB signaling to limit epithelial inflammatory response to kidney injury**

Xuan Peng^1,2,3§^, Yating Wang^1,2,3§^, Huiyan Li^1,2,3^, Jinjin Fan^1,2,3^, Jiani Shen^1,2,3^, Xueqing Yu^1,2,3^, Yi Zhou^1,2,3 *^, Haiping Mao^1,2,3 *^

^1^ Department of Nephrology, the First Affiliated Hospital, Sun Yat-Sen University;

^2^ NHC Key Laboratory of Nephrology

^3^ Guangdong Provincial Key Laboratory of Nephrology, Guangzhou, 510080, China

**Supplementary figure legends**

**Supplementary Fig. 1 *ATG5* deficiency promotes inflammatory cytokines expression. a** The mRNA expression of IL-1β, IL-6, and TNF-α in kidney tissues were determined by real-time PCR. Data are means ± SEM (n = 6). *, *P* < 0.001 vs. corresponding sham; #, *P* < 0.05 vs. corresponding *ATG5^+/+^* mice after UUO. **b** The mRNA levels of IL-1β, IL-6 and TNF-α in HK-2 cells transfected with either scramble or *ATG5* siRNA followed by treatment of Ang II for 24 h. Data are means ± SEM (n = 3). *, *P* < 0.001 vs. Ang II-untreated cells; #, *P* < 0.05 vs. Ang II-treated cells with scramble siRNA transfection. **c** The mRNA levels of IL-1β, IL-6, and TNF-α in primary *ATG5*^+/+^ and *ATG5*^-/-^ TECs. Data are means ± SEM (n = 3). *, *P* < 0.001 vs. Ang II-untreated cells; #, *P* < 0.05 vs. Ang II-treated *ATG5*^+/+^ cells.

**Supplementary Fig. 2 NF-κB signaling is activated by AngII in HK-2 cells.** **a** Serum-deprived HK-2 cells were stimulated with Ang II for the indicated time period and cell lysates were probed with antibodies against p-p65, p65, and β-actin. **b** Densitometry of p-p65 proteins in immunoblots (relative to β-actin). Data are means ±SEM (n =3); *, *P*<0.01 vs. control group.

**Supplementary Fig. 3** **NF-κB activation promotes tubule epithelial cells arrested at the G2/M phase of the cell cycle after injury.** **a** Representative immunostaining of p-p65 (red), p-H3 (green) and DAPI (blue) in kidney sections. Scale bar: 20 μm. **b** Representative immunostaining of IL-1β (red), p-H3 (green) and DAPI (blue) in kidney sections. Scale bar: 20 μm. **c** HK-2 cells treated with Ang II for 24 h and immunofluorescence staining for p-p65 (red) and p-H3 (green). Cell nuclei were counterstained with DAPI (blue). **d** HK-2 cells were pretreated with or without JSH-23, followed by exposure to Ang II for 48 h. Cell cycle analysis was performed among different groups by flow cytometry. Data are mean ± SEM (n = 3); **P*<0.05 vs. Ang II-untreated cells; #*P*<0.05 vs. cells with Ang II-treated alone.
